# Peri-operative physiotherapy

**DOI:** 10.1186/2049-6958-8-4

**Published:** 2013-01-23

**Authors:** Dewi Nurul Makhabah, Federica Martino, Nicolino Ambrosino

**Affiliations:** 1 Pulmonary Rehabilitation and Weaning Center, Auxilium Vitae, Volterra, Italy; 2 Pulmonary Unit, University Hospital, Pisa, Italy; 3 Respiratory Unit, Cardio-Thoracic Department University Hospital, Pisa, Italy

## Abstract

Postoperative pulmonary complications (PPC) are a major cause of morbidity, mortality, prolonged hospital stay, and increased cost of care. Physiotherapy (PT) programs in post-surgical and critical area patients are aimed to reduce the risks of PPC due to long-term bed-rest, to improve the patient’s quality of life and residual function, and to avoid new hospitalizations. At this purpose, PT programs apply advanced cost-effective therapeutic modalities to decrease complications and patient’s ventilator-dependency. Strategies to reduce PPC include monitoring and reduction of risk factors, improving preoperative status, patient education, smoking cessation, intra-operative and postoperative pulmonary care. Different PT techniques, as a part of the comprehensive management of patients undergoing cardiac, upper abdominal, and thoracic surgery, may prevent and treat PPC such as secretion retention, atelectasis, and pneumonia.

## Review

A postoperative pulmonary complication (PPC) is defined as any pulmonary abnormality occurring during the postoperative period and resulting in clinically significant disease or dysfunction, adversely affecting the clinical course [1]. The respiratory system is directly influenced by the type of surgery, the organ involved and the method used. Postoperative pulmonary complications are a major cause of morbidity, mortality, prolonged hospital stay, and increased cost of care [2]. They may be associated to respiratory muscle dysfunction which may start at induction of anesthesia and continue into the postoperative period. As a matter of fact, the surgical lesion, possible involvement of the phrenic nerve, drugs for sedation, all can lead to ventilation derangement. As a consequence a reduction in functional residual (FRC) and vital (VC) capacity can be observed resulting in higher likelihood of infection [2,3].

Patients with chronic obstructive pulmonary disease (COPD) may be at particular risk of PPC, up to 30% of these patients possibly developing PPC after thoracic surgery [1,4]. The postoperative course of patients with severe emphysema may take advantage from a multidisciplinary management including preoperative smoking cessation, drug therapy optimisation, physiotherapy (PT) programs, early mobilization and early extubation [5,6].

Physiotherapy programs in post-surgical and critical area patients are aimed to reduce the risks of PPC due to long-term bed-rest, to improve the patient’s quality of life (QoL) and residual function, and to avoid new hospitalizations. At this purpose, PT programs apply advanced cost-effective therapeutic modalities to decrease complications and patient’s ventilator-dependency [7,8].

Strategies to reduce PPC include monitoring and reduction of risk factors, improving preoperative status, patient education, smoking cessation, intra-operative and postoperative pulmonary care. Different PT techniques, as a part of the comprehensive management of patients undergoing cardiac, upper abdominal, and thoracic surgery, may prevent and treat PPC such as secretion retention, atelectasis, and pneumonia [9-12]. Patients undergoing PT in the immediate postoperative period have likely reduced prevalence of “difficult weaning” and reduced mobility. In addition, QoL, Health status and general conditions may benefit [13,14].

An important component of the weaning protocols should be PT availability. Weaning protocols and PT are related interventions to shorten patient’s recovery period. Both are used also in uncooperative and bedridden critically ill patients [15,16]. Early PT in patients with surgery-associated critical illness may have significant impact on physical and functional outcomes in addition to decreasing length of ICU stay and its associated resource implications. The potential for early PT should therefore be assessed immediately upon surgical ICU admission in all patients and continue throughout the acute admission [17].

## Physiotherapy modalities

### Early mobilization

Prolonged immobility is a main cause of muscle weakness in patients admitted to ICU, conversely early PT has an important role in the recovery of these patients. Early mobilization and muscle training can improve functional outcomes, cognitive and respiratory conditions, reducing the risks of venous stasis and deep vein thrombosis. For this reason the exercise in the early stages after surgery, when possible, can be helpful in preventing PPC such as atelectasis, reducing the need of painkillers, improving recovery and avoiding neuromuscular complications [6,7,13,14,18-20].

Suggested goals of early mobilization, although not always demonstrated, may be [21]:

1. Increase in lung volumes, optimization of ventilation to perfusion ratio (VA/Q) and better airway clearance; 2. Reduction of risk of immobility associated with PPC; 3. Improvement in level of consciousness; 4. Increase in functional independence; 5. Improvement in cardiovascular fitness; 6. Psychological benefit.

### Respiratory muscle training

Respiratory muscle weakness, imbalance between muscle strength and the load of the respiratory system and cardiovascular impairment are major determinants of weaning failure in ventilated patients, including post-surgery. In ICU patients these factors and the excessive use of controlled mechanical ventilation may lead to rapid diaphragmatic atrophy and dysfunction [22]. In many studies high risk patients waiting for elective coronary artery bypass graft (CABG) surgery get a benefit from preoperative PT programs including respiratory muscle training. A reduction of PPC and hospital stay was observed in patients undergoing respiratory muscle training compared to controls [23-25]. Nomori et al. [26] showed that inspiratory muscle training before thoracic surgery may prevent PPC. A systematic review of studies on ICU ventilator-dependent COPD patients showed that inspiratory muscle training significantly increased inspiratory muscle strength. However this study failed to clarify whether the increase in inspiratory muscle strength may lead to a shorter duration of mechanical ventilation, if it improved weaning success, or improved survival. Further large randomized studies are required to clarify the impact of inspiratory muscle training on patients receiving mechanical ventilation [27].

### Neuromuscular electrical stimulation

Neuromuscular electrical stimulation (NMES) can induce changes in muscle function without any form of ventilatory stress [28]. NMES can be easily performed in the ICU, applied to lower limb muscles of patients laying in bed. Nevertheless, to date, no clinical studies have yet completely demonstrated the additional effect of NMES on exercise tolerance when compared with conventional training. A study suggests that both conventional chest physiotherapy and conventional chest physiotherapy + transcutaneous electric diaphragmatic stimulation prevent the reduction of pulmonary function during the Roux-en-Y gastric bypass postoperative period, and that transcutaneous electric diaphragmatic stimulation also contributes to expiratory muscle strength [29]. A small study reported that transcutaneous electrical nerve stimulation (TENS) was considerably active in reducing pain, and the increase in respiratory muscle strength at first-day after CABG surgery [30]. A randomized study confirmed that TENS is a valuable approach to reduce post-thoracotomy pain with decrease of cytokine production and of analgesic intake, and with positive effects on pulmonary ventilation function [31].

### Breathing exercises

In a meta-analysis of Thomas et al. there was a benefit from the use of Incentive Spirometry (IS) and breathing exercises after abdominal surgery. It remains, however, unclear the benefit on the incidence of some PPC as atelectasis and infection [32]. Carvalho et al [33] performed a systematic review to evaluate the evidence of the use of IS for the prevention of PPC and for the recovery of lung function in patients undergoing abdominal, cardiac and thoracic surgeries. There was no evidence to support the use of IS in the management of surgical patients. Despite this, the use of IS remains widely used without standardisation in clinical practice.

Other meta-analysis were conducted to evaluate the effectiveness of the PT in preventing the PPC. In a study of Overend et al. [34] patients undergoing cardiac or abdominal surgery did not seem to get any help from IS. In a systematic review Pasquina et al. [35] have assessed whether respiratory PT prevents PPC after cardiac surgery evaluating 18 trials. Anyway most of them were of low quality. They tested physical therapy (13 trials), IS (8), continuous positive airway pressure (CPAP:5), and intermittent positive pressure breathing (IPPB: 3). The authors did not show any advantage from the practice of PT. A few years later Pasquina et al. [36] found no evidence for the application of PT routine in patients undergoing abdominal surgery.

## Fast-track rehabilitation

Fast-track rehabilitation programs include easy methods that have the potential to decline morbidity, hospital stay, and increase pain control when compared with conventional care. Patients with lung cancer who undergo lobectomy can be treated with fast-track rehabilitation. Fast-track rehabilitation is based on: minimally invasive surgical techniques using video-assisted and muscle sparring incisions, normovolemia, normothermia, good oxygenation, euglicaemia, no unnecessary antibiotics, epidural patient-controlled analgesia, systemic opiods-free analgesia, early ambulation and oral feeding [37].

## Thoracic surgery

Patients undergoing upper abdominal and thoracic surgery have a decreased postoperative VC, which leads to VA/Q mismatch and contributes to development of hypoxaemia. Thus, the incidence rate of PPC is substantially higher for thoracic and upper abdominal surgery than for lower abdominal surgery. This can be explained by diaphragmatic dysfunction.

Clinical outcomes and costs of 119 patients undergoing lobectomy**,** who underwent incentive spirometry as well as chest PT**,** and 520 historical control subjects who did not receive this care**,** were reviewed by Varela et al. [38]. The length of stay, the number of atelectasis, and the cost in the PT group were significantly less than in the control group. Furthermore**,** a review by Orman and Westerdahl demonstrated a little gain with positive expiratory pressure (PEP) treatment versus other physiotherapy breathing techniques in patients who underwent thoracic surgery [39].

A reduction in exercise tolerance and QoL is frequently observed, as a consequence of a lobectomy. In a randomized study of Arbane et al. [40] 53 patients with lung cancer were divided into two groups. Every patient underwent a thoracotomy, but one group of patients was treated with a PT program. No difference in QoL was reported between the two groups and no difference was detected in distance covered at 6 minute walking test even if the muscle strength of lower limb was higher in the group treated with physiotherapy. However**,** exercise capacity returned to pre-operative levels after 12 weeks independently of additional support offered.

## Abdominal surgery

Orman and Westerdahl in 2010 reviewed 6 randomized controlled trials that used PEP in adults who underwent abdominal surgery. They found out that just one trial showed a benefit for patients treated with PEP versus other PT breathing techniques [39].

The usefulness of IS in preventing PPC has not been confirmed by a recent meta-analysis on patients undergoing upper abdominal surgery [41]. Lunardi et al. found that PPC after oesophagectomy are reduced in patients who had undergone chest PT before surgery. Nevertheless the hospital length of stay did not differ in patients treated with PT compared to control patients [42]. Several reviews evaluated the potential effects of chest PT in the pre- and post-operative time. Manzano et al. found on a small sample of patients [43] that chest PT after abdominal surgery can improve oxygen haemoglobin saturation without worsening abdominal pain.

A recent systematic review [44] analyzed the effect of PT to prevent PPC in patients undergoing upper abdominal surgery. They excluded studies on patients with comorbidities and undergoing IS and mechanical ventilation more than 48 hours. Patients treated also with breathing exercises showed a greater respiratory muscles strength than control patients. No difference was observed in the PPC incidence between PT group versus control group. Nevertheless the mean quality of the studies included in the review was low.

Valknet et al. [45] in a systematic review demonstrated a benefit from PT before cardiac and abdominal surgery. In this review patients treated before surgery with PT had a reduced number of PPC and length of hospital stay. Nevertheless, further and systematic studies are required to corroborate these encouraging studies and to avoid equivocal interpretations [46].

## Cardiac surgery

Interventions such as breathing and coughing exercises, early ambulating, and pulmonary clearing techniques are often used by physical therapists to prevent PPC after CABG surgery. However, there is controversy concerning both the efficacy of these postoperative procedures in reducing the incidence of PPCs and the proper strategy for the identification of patients who might benefit from such interventions. In contrast to the controversy that exists relative to patients undergoing general surgery, similar procedures performed before CABG surgery were shown to be effective and able to lower the risk of PPCs. Evidence derived from small trials suggests that preoperative PT reduces PPC (atelectasis and pneumonia) and length of hospital stay in patients undergoing elective cardiac surgery. There is a lack of evidence that preoperative PT reduces postoperative pneumothorax, prolonged mechanical ventilation or all-cause deaths [47-49].

Castillo and Haas [50] have shown the effectiveness of pre- and post-operative PT in reducing significantly the number of patients who develop atelectasis after surgery. On the contrary, they did not show any benefit in reducing respiratory infections. Robinson et al. [51] and Felcar et al. [52] evaluated the potential benefit of pre- and post-operative PT in a selected group of patients after cardiac surgery. The first authors analyzed severe COPD patients while the others analyzed a pediatric population. They found comparable results: PPC were less when PT was performed also before surgery. Yanez-Brage et al. also found a lower number of pulmonary atelectasis in patients who underwent a respiratory PT before surgery of CABG [12]. Valkenet in a systematic review described a benefit of preoperative respiratory PT in the number of PPC and in hospital stay both after cardiac or abdominal surgery [45]. Furthermore, when the PT program aimed at early mobilization is tailored to the age, needs and possible pathologies of the patient, best results are found in recovery after surgery [53]. It has been recently shown that inspiratory muscle training may improve inspiratory muscle strength and increase paralyzed diaphragm mobility after major cardiac surgery [54]. There is no evidence that a specific modality of exercise, either walking or cycling can influence the outcome of PT in these cases [55].

## Lung cancer

Lung cancer patients seem to be a good population to undergo a PT program [56]. For this reason the European Respiratory Society (ERS) and the European Society of Thoracic Surgeons (ESTS) proposed and published evidence-based guidelines on the use of PT programs in lung cancer patients after lung resection [57]. The conclusions were that current evidence suggests that exercise training, an intervention able to improve exercise tolerance, may improve surgical risk and/or recovery, symptom control, and possibly, risk of dying following a lung cancer diagnosis. Exercise training is acknowledged as one of the most effective interventions to improve exercise tolerance [58]. Patients with lung cancer who underwent to a program of perioperative intensive chest PT seem to have a less overall pulmonary morbidity after surgery [59].

Park et al. evaluated the effects of high frequency chest wall oscillation therapy after pulmonary lobectomy in patients with non-small cell lung cancer (NSCLC) compared to conventional chest PT. In patients who underwent high frequency chest wall oscillation therapy there was a better and quicker recovery of pulmonary function than patients who were treated with conventional chest PT. No side effects were highlighted [60]. Pehlivan et al. observed 60 patients with NSLCL waiting for surgery and divided them into two groups. Every patient was treated with a PT program, but one of the two groups was treated with an intensive rehabilitation program while the other group with a conventional one. Patients undergoing intensive rehabilitation showed a better blood gas exchange, VA/Q and a reduction in hospital stay [61]. Nagarajan et al. in 2011 evaluated literature about preoperative PT for patients waiting for a lung resection. They concluded that many studies show a gain in exercise capacity and a better maintenance of pulmonary function, but they do not find a certain gain in the number of PPC [62].

## Conclusions

There are many evidences that the number of PPC after abdominal surgery and open-heart surgery is reduced by preoperative PT programs. The preoperative PT results in a reduction of radiographic changes, a modification of objectivity chest, an improved gas exchange as well as improved QoL and a decrease in hospital stay [63-66]. However, at present no PT treatment has been identified as more effective than others.

The evidence of the effectiveness of preoperative physiotherapy protocols in reducing PPC in critically ill patients [67] is weak.

## **Competing interests**

The authors declare that they have no competing interests.
